# Relapse in leprosy and drug resistance assessment in a tertiary hospital of the state of Espírito Santo, Brazil

**DOI:** 10.1590/0037-8682-0375-2020

**Published:** 2021-03-08

**Authors:** Danielle Ferreira Chagas, Lucia Martins Diniz, Elton Almeida Lucas, Milton Ozório de Moraes

**Affiliations:** 1 Universidade Federal do Espirito Santo, Hospital Universitário Cassiano Antônio Morais, Serviço de Dermatologia, Vitória, ES, Brasil.; 2 Fundação Oswaldo Cruz, Serviço de Hansenologia, Rio de Janeiro, RJ, Brasil.

**Keywords:** Leprosy, Recurrence, Relapse, Epidemiology, Drug resistance

## Abstract

**INTRODUCTION:**

Leprosy recurrence is the reappearance of the disease after treatment with current schemes and discharged for cure and may have variable incubation periods.

**METHODS:**

This is a descriptive observational study of leprosy recurrence in Espírito Santo diagnosed between January 2018 and January 2020.

**RESULTS:**

One hundred and ninety-two cases were available, of which 30 were diagnosed with leprosy recurrence.

**CONCLUSIONS:**

In 25 cases, the incubation period was 5-15 years after the first treatment, favoring bacillary persistence. In the remaining 5 cases, the disease had recurred after 15 years, pointing to reinfection as none of them exhibited drug resistance.

In 1981, the World Health Organization recommended multidrug therapy (MDT) for treating leprosy. As a result, the prevalence of the disease has decreased worldwide^1^ ^,^ ^3^. However, thousands of new cases are observed in Brazil every year. In 2018, the detection rate was 13.2 new cases per 100,000 inhabitants^2^. With respect to the State of Espírito Santo, in that same year, the average detection rate of new leprosy cases was 11.48 cases per 100,000 inhabitants, the highest in the entire region^2^.

Leprosy recurrence is diagnosed in every patient treated regularly with standardized official regimens, discharged for cure, and after a variable incubation period, usually three years in paucibacillary cases (PB), and five years in multibacillary cases (MB), they exhibit clinical signs and symptoms of infection again^3^.

The diagnosis of recurrence, as standardized by the Ordinance of the Ministry of Health, Nº. 15 of 2015, should meet clinical and laboratory criteria, that is, bacilloscopy index (BI), histopathological tests of skin lesions, and testing drug resistance via the extraction of bacillus DNA through molecular biology^4^.

The predisposing factors that influence the pathogenesis of recurrence are: a) bacillary persistence, present in about 10% of MB cases, more frequent in patients with a high BI, b) drug resistance, due to some mutation in the drug target genes; and, c) reinfection, possibly resulting from the hyperendemicity of the area^4^.

The goal of the present study was to describe the clinical and epidemiological characteristics of leprosy recurrence cases diagnosed at a reference service for leprosy recurrence in the state of Espírito Santo, Brazil.

This is an observational, descriptive study of leprosy recurrence cases notified from January 2018 to January 2020 in the State of Espírito Santo, Brazil. The following strategies were used to assess these cases:

1. Identification of all cases diagnosed with suspected leprosy recurrence referred to the Reference Hospital of Espírito Santo.
2. Assessment of the following variables: age, sex, incubation period, clinical forms, BI, and therapeutic indexes in the first diagnosis and in the recurrence, and the results of histopathological examinations and drug resistance in cases of leprosy recurrence.

The inclusion criteria were as follows: patients referred with clinical suspicion of leprosy recurrence, who had been treated for this disease on a regular basis by the official regimes, discharged for cure, and exhibited clinical signs and symptoms indicating the recurrence of the disease. Exclusion criteria were as follows: diagnostic error, irregularity in the first treatment, leprosy reaction, and other dermatological diagnoses.

In case of suspicion of leprosy recurrence, two biopsies of skin lesions were performed: one for histopathological studies and the other for molecular biological drug resistance assessment to be performed at a reference laboratory molecular biology. The slides were subsequently processed to extract genomic DNA, and polymerase chain reactions (PCR) were performed to amplify the drug-resistance determining region of *rpoB*, *folP1*, and *gyrA* genes, specific target regions for rifampicin, dapsone, and ofloxacin, respectively.

Once the infections had been cured, the patients’ data were statistically analyzed using IBM SPSS Statistics version 21.0 software. 

Patient identification data were kept confidential. The project was approved by the Research Ethics Committee of the Health Sciences Center under Protocol Nº. 3768024.

One hundred and ninety-two suspected cases of leprosy recurrence were referred to the university hospital from January 2018 to January 2020. These patients were from several municipalities in the state of Espírito Santo. Of the total number of patients, 162 were excluded because they did not fit the diagnosis of recurrence. Therefore, 30 (15.6 %) patients were included with the diagnosis of leprosy recurrence.

Regarding the 30 cases with recurrence, the ages ranged from 23 to 72 years, with an average age of 50 years and 5 months, and a median of 51 years (standard deviation = 10 years and 3 months). Among these patients, 17 (56.6%) were men and 13 (43.4%) were women.

The assessment of the incubation period indicated that 20 patients (66.7%) had relapsed after 5 to 10 years of clinical cure, five patients (16.7%) from 10 to 15 years, and 5 (16.7%) patients after 15 years (Table 1). The average incubation period was 10 years and 7 months (minimum of 5 years and maximum of 27 years).

**TABLE 1:** Incubation period of relapse in relation to the clinical forms of leprosy.

Clinical classification of leprosy	Incubation period of leprosy recurrence (years)					
	≥5-10	%	10-15	%	≥15	%
Borderline-borderline	10	33.3	0	0	02	6.7
Borderline-lepromatous	05	16.7	2	6.7	0	0
Lepromatous	05	16.7	3	10	03	10
**Total**	**20**	**66.7**	**5**	**16.7**	**5**	**16.7**

The distribution of patients according to the clinical forms of the disease in the first diagnosis and in the recurrence is shown in Table 2.

**TABLE 2:** Classification of clinical forms of leprosy in the first diagnosis and in the relapse.

Classification of leprosy	First diagnosis		Relapse	
	N	%	N	%
Borderline-tuberculoid	04	13.3	0	0
Borderline-borderline	13	43.3	13	43.3
Borderline-lepromatous	2	6.6	7	23.3
Lepromatous	06	20.0	10	33.3
Indeterminate	04	13.3	0	0
Tuberculoid	01	3.3	0	0
**Total**	**30**	**100**	**30**	**100**

The BI assessment indicated that 19 patients (63.3%) had positive bacilloscopy with intact bacilli (ranging from 1.0 to 5.5) at the first diagnosis and 19 patients (63.3%) (ranging from 1 to 5.25) had a recurrence. Five patients who were initially negative had relapsed. Five patients had positive bacilloscopy at the first diagnosis, and it was negative at recurrence. 

All patients underwent biopsies, and the material was sent for histopathological studies, proving the occurrence of the disease in 23 patients (76.6%) and the results of 7 were inconclusive (23.4%). The patients were distributed according to clinical-histopathological forms (Table 3). Twenty patients were acid-fast bacilli smear-positive, varying between one and six crosses, some showing globi formation.

**TABLE 3:** Results of histophatological examinations after relapse.

Results of histophatological	N	%
Inconclusive	07	23.3
Tuberculoid	03	10
Borderline-borderline	05	16.6
Borderline-lepromatous	05	16.6
Lepromatous	10	33.3
**Total**	**30**	**100**

In the assessment of drug resistance using PCR, the tests were negative for *rpoB* genes in codons 441 (516) G>A, 451 (526) C>T, 456 (531) C>T,G, and 458 (533) T>C. They were also negative for the *folP* gene in codons 53 (A>G and C>T) and 55 (C>T), and for the *gyrA* gene in codons 89 (G>T), 91 (C>T), and 92 (T>C).

Ten patients received MDT/PB regimens in the first diagnosis and 20 patients received MDT/MB correctly and regularly. In the recurrence group, all were treated with PCT/MB and responded favorably, after which they were discharged from the hospital. The use of alternative treatments in three patients with MB in the first diagnosis, and in six patients with recurrence was due to side effects caused by dapsone, which was replaced by ofloxacin. 

During the incubation period, of the 30 patients, 12 (40%) reported that the intradomiciliary contacts had leprosy.

A total of 208,619 new leprosy cases were reported in 2018 in 127 countries, indicating continuous transmission by foci of active infection^3^. One of the strategies established for monitoring the success achieved by leprosy control programs is the detection of the factors involved in leprosy recurrence, which was the goal of the present study.

Regarding the 30 patients diagnosed with leprosy recurrence, the mean age was 50 years and 5 months (range, 23 to 72 years). This result is in line with those of studies conducted by Ferreira et al^5^. and Shen et al^6^. It is expected that recurrence will generally occur in individuals aged over 50 years, as it depends on the first episode of infection and regular treatment, associated with the long incubation period for the reappearance of the disease due to the slow and gradual multiplication of *Mycobacterium leprae*.

In the present study, men were the most affected by leprosy recurrence (17/56.6%), as also observed in other studies on the recurrence of infection^5^ ^-^ ^7^ and in the Brazilian epidemiological bulletin of 2020^2^. Prabu et al. (2015)^8^ studied 58 patients with recurrence and observed a rate of 7.7 men/1,000 individuals per year, in comparison to 4.2 women/1,000 individuals per year.

The clinical form of recurrence most frequently diagnosed in the present study was borderline (70%). The studies conducted by Diniz et al. (33.7%)^7^, Ferreira et al. (39.6%)^5^, and Shen et al. (55%)^6^ found that the borderline form was the most susceptible to recurrence. These authors referred to the immune instability present in this form, which contributed to the recurrence of the disease. Guerrero-Guerrero et al^9^ *.* observed that the virchowian form was four times more likely to occur in comparison to other clinical forms.

Shetty et al.^10^ studied 62 cases of leprosy recurrence in India after several therapeutic regimens. These authors observed that 47% of the cases had the same clinical aspects of recurrence. They added that there was no evolution of any borderline-virchowian case into borderline-tuberculoid cases; however, the opposite occurred in 15 cases (24%), demonstrating decreased immunity in the patients. In our study, 9 cases (30%), considered as PB in the first diagnosis evolved clinically to MB, showing immune deterioration.

In our assessment and during the literature review, we generally did not consider a time interval of less than five years in cases of leprosy recurrence as recurrence cases, but that it was associated with inadequate therapy and errors of operational classification at the time of the first diagnosis. The authors emphasized the importance of following-up patients after the end of treatments, at least during the first five years, in order to diagnose recurrence cases earlier.

When the time interval is between five and fifteen years, the mechanism involved is probably bacillary persistence, which can occur due to the adaptive capacity of *M. leprae* in remaining inactive in immunologically inappropriate places under conditions of low metabolism during treatment. It can recover its active metabolic form some time after the end of therapy, thus showing new signs of the disease^1^. Twenty-five (83.3%) cases assessed in the present study had relapsed during this period, with an average of 10 years and seven months. This finding was similar to those of the studies conducted by Balagon et al.^11^

Leprosy recurrence cases that occur with long intervals between the first and second diagnoses, usually > 15 years, are mainly observed in patients continuously exposed to the bacillus^1^. In our study, 12 (40%) patients had reported intradomiciliary contact with patients diagnosed with leprosy, which could explain recurrences resulting from hyperendemicity of the area or reinfection^10^ ^-^ ^12^.

In a study conducted by Shen et al.^7^, of the 40 patients studied with recurrence, 13 (32.5%) had family members who had exhibited the infection during the incubation period of recurrence. Stefani et al.^12^ studied three cases of leprosy recurrence and found differences in DNA sequence between the bacillus strains in the first and second diagnoses of leprosy in these patients, which indicates a great possibility of reinfection of the disease.

Ferreira et al.^5^ assessed 53 patients with leprosy recurrence between 2005 and 2007 and performed biopsies for histopathological studies in 26 patients (49%). Shen et al.^6^ assessed 40 cases of recurrence and diagnosed the disease in 23 cases (57.5%) based on histopathological examinations. These results are different from those of ours, given that in the present study, 76.6% of the patients had the disease proven by histopathological examinations.

In the present study, all cases assessed through molecular biology were negative for drug resistance, corroborating the absolute effectiveness of the therapeutic scheme proposed by the Brazilian Ministry of Health^4^. However, some studies have indicated drug resistance to rifampicin, dapsone, and ofloxacin, also in cases of leprosy recurrence^12^ ^-^ ^14^, in addition to reporting mutations other than the traditional ones, such as *nth* (DNA repair), *rpo*, *rpoC* (rifampicin), *gyrB* (ofloxacin), and *23s rRNA* (clarithromycin), but still having uncertain roles^13^. Reja et al.^14^ and Chauffour et al.^15^affirmed that even cases of negative bacilloscopy can be targets of drug resistance, deserving even more attention during the follow-up period.

In conclusion, the present study indicated that leprosy recurrence in the 30 cases assessed was more frequent in MB cases of male patients aged over 50 years. Regarding the possible causes of recurrence, in 25 cases, the incubation period ranged from 5 to 15 years after the end of the first treatment, favoring bacillary persistence. In the remaining 5 cases, the patients had relapsed after 15 years. This fact indicated reinfections resulting from the hyperendemicity of the area, given that none of the cases were characterized by drug resistance found by molecular biology tests for the *folP1*, *rpoB*, and *gyrA* genes.

Understanding the mechanisms underlying leprosy recurrence will promote the implementation of interventions aimed at reducing the bacillary burden in exposed populations. In addition, it is essential to strengthen the follow-ups of patients after treatment in order to detect possible recurrence episodes early.
